# Predictive Value of [^18^F]FDG PET/CT for Lymph Node Metastasis in Rectal Cancer

**DOI:** 10.1038/s41598-019-41422-8

**Published:** 2019-03-21

**Authors:** Sung Hoon Kim, Bong-Il Song, Beong Woo Kim, Hae Won Kim, Kyoung Sook Won, Sung Uk Bae, Woon Kyung Jeong, Seong Kyu Baek

**Affiliations:** 1 Department of Nuclear Medicine, Dongsan Medical Center, Keimyung University School of Medicine, Daegu, Korea; 2 Department of Surgery, Dongsan Medical Center, Keimyung University School of Medicine, Daegu, Korea

## Abstract

[^18^F]Fluorodeoxyglucose ([^18^F]FDG) Positron emission tomography/computed tomography (PET/CT) is commonly used for rectal cancer staging, but improved diagnostic methods for nodal metastases are needed. We aimed to evaluate whether the combination model of the metabolic tumor volume of primary tumor (T_MTV) and maximum standardized uptake value of lymph node (N_SUVmax) on pretreatment [^18^F]FDG PET/CT could improve nodal metastases prediction in rectal cancer. We enrolled a total of 166 rectal cancer patients who underwent pretreatment [^18^F]FDG PET/CT and surgical resection without neoadjuvant treatment between January 2009 and August 2016. Visual and semiquantitative PET/CT parameters were obtained. Associations between clinicopathological, PET/CT-derived variables and nodal metastases were evaluated by logistic regression analysis. Nodal metastases were confirmed histologically in 68 of the 166 patients (41%). Uni- and multivariate analyses demonstrated T_MTV and N_SUVmax were independent predictive factors for nodal metastases. The c-statistics of the combination model was 0.806 (Standard Error, 0.034; 95% Confidence Interval, 0.737–0.863), which showed significant improvement compared to T_MTV (0.698, *P* = 0.0002) or N_SUVmax (0.720, *P* = 0.0008) alone. T_MTV and N_SUVmax are independently correlated with nodal metastases. Furthermore, the combination model showed improved performance for risk prediction; thus, [^18^F]FDG PET/CT might have a role in rectal cancer staging and treatment planning.

## Introduction

Colorectal cancer (CRC) is one of the most common malignancies, and it is the second leading cause of death from cancer in the United States. It is estimated that 135,430 new cases developed and 50,260 deaths occurred in 2017^1^. Identifying lymph node (LN) metastases is one of the most important steps for staging rectal cancer because it helps guide therapeutic decisions and determine the long-term outcome^2,3^.

Clinically, LN size, measured by computed tomography (CT) or magnetic resonance imaging (MRI), is the most common criteria used to determine pathologic LNs. An LN with greater than 1 cm short-axis diameter is considered pathologic; however, the upper limit of benign LNs differs depending on the anatomic location and tumor type^4,5^. Furthermore, nodal metastases can occur in normal-sized LNs less than 1 cm^6^. Thus, nodal staging based on LN size shows low sensitivity in identifying small metastatic LNs in patients with rectal cancer using conventional imaging modalities^7,8^.

Positron emission tomography (PET)/CT with [^18^F]Fluorodeoxyglucose ([^18^F]FDG) has been widely used for staging, restaging, and detection of recurrent disease in rectal cancer^9–11^. Although the specificity of [^18^F]FDG PET/CT in detecting nodal metastases has been known to be excellent, its sensitivity is relatively low^12^. Nodal [^18^F]FDG uptake findings alone could play a strong independent predictive factor for LN metastases. If the hypermetabolic LN, which show high [^18^F]FDG uptake, is observed in [^18^F]FDG PET/CT, this strongly suggests LN metastases, but not all hypermetabolic LNs are LN metastases. Normal vascular structures such as venous flexus or inflammatory reactive LNs were sometimes misinterpreted as metastatic LNs in CRC^12,13^. Although, strict criteria for the diagnosis of LN metastases in [^18^F]FDG PET/CT could reduce false-positive rates but decrease the sensitivity. Thus, sole use of nodal [^18^F]FDG uptake finding is imperfect for identifying LN metastases, and better predictive tools are needed.

Recently, metabolic tumor volume (MTV), as a volumetric parameter of PET/CT, has been suggested to be a predictive marker of survival outcomes in CRC^14,15^. Previous studies show that MTV could be a promising predictor for LN metastasis^16–18^; however, no studies have attempted to develop a prediction model for LN metastases status in rectal cancer using both MTV of the primary tumor (T_MTV) and [^18^F]FDG-avid LN findings.

The purpose of this retrospective study was to determine whether the combination model of T_MTV and maximum standardized uptake value (SUVmax) of the LN (N_SUVmax), measured by [^18^F]FDG PET/CT, could improve the prediction of LN metastases in patients with rectal cancer.

## Results

### Patient Characteristics

A total of 166 patients with rectal cancer who received curative surgical resection were retrospectively analyzed. The characteristics of the enrolled patients (mean age, 66.7 ± 10.6 years) and the associations with LN metastases are listed in Table 1. LN metastases were confirmed histologically in 68 patients (41%), and 98 patients (59%) presented with no LN metastases. Although the majority of patients were diagnosed with 7th American Joint Committee on Cancer (AJCC) stage I–III cancer, two patients with pathologic stage Tis were classified as stage 0, and one patient with distant metastasis was classified as stage IV. The stage IV patient had 6 nodal metastases (N2a) and underwent a low anterior resection of a single hepatic metastasis and left hepatectomy. Pathologic stages according to the 7th AJCC and PET parameters, such as SUVmax of primary tumor (T_SUVmax), T_MTV, and N_SUVmax, were significantly different between the two groups; however, no significant differences were found with respect to pre-operative carcinoembryonic antigen (CEA), pathologic tumor size, and histologic grade. Thirty-nine of the 166 patients (23.5%) showed positive nodal uptake by [^18^F]FDG PET/CT findings. Of these 39 patients, 33 (84.6%) had histologically confirmed LN metastases. On the contrary, LN metastases were confirmed histologically in 35 (27.6%) of the 127 patients with negative nodal FDG uptake. For detecting LN metastases, [^18^F]FDG PET/CT had a sensitivity of 48.5%, a specificity of 93.9%, a positive predictive value of 84.6%, and a negative predictive value of 72.4%.

**Table 1:** Patient Characteristics. CEA = Carcinoembryonic Antigen; AJCC = American Joint Committee on Cancer (7th ed.); PET/CT = Positron Emission Tomography/Computed Tomography; SUVmax = maximum standardized uptake value; MTV = Metabolic Tumor Volume; *Grade cannot be assessed in three patients with mucinous carcinoma.

Variables	All patients (N = 166)	LN metastasis (−) (N = 98)	LN metastasis (+) (N = 68)	P value
Age, years	66.7 ± 10.6	67.1 ± 9.3	66.0 ± 12.2	0.560
Sex				0.202
Male	94 (56.6%)	60 (61.2%)	34 (50.0%)	
Female	72 (43.4%)	38 (38.8%)	34 (50.0%)	
Pre-operative CEA, ng/ml	5.7 ± 21.8	6.1 ± 27.0	5.1 ± 11.0	0.757
Pathologic tumor size, cm	4.6 ± 5.4	4.3 ± 6.8	5.0 ± 2.2	0.362
Pathologic T stage				<0.001
Tis	2 (1.2%)	2 (2.0%)	0 (0.0%)	
T1	25 (15.1%)	24 (24.5%)	1 (1.5%)	
T2	44 (26.5%)	34 (34.7%)	10 (14.7%)	
T3	77 (46.4%)	34 (34.7%)	43 (63.2%)	
T4	18 (10.8%)	4 (4.1%)	14 (20.6%)	
Pathologic N stage				<0.001
N0	98 (59.0%)	98 (100.0%)	0 (0.0%)	
N1	40 (24.1%)	0 (0.0%)	40 (58.8%)	
N2	28 (16.9%)	0 (0.0%)	28 (41.2%)	
AJCC stage				<0.001
0	2 (1.2%)	2 (2.0%)	0 (0.0%)	
I	58 (34.9%)	58 (59.2%)	0 (0.0%)	
II	38 (22.9%)	38 (38.8%)	0 (0.0%)	
III	67 (40.4%)	0 (0.0%)	67 (98.5%)	
IV	1 (0.6%)	0 (0.0%)	1 (1.5%)	
Histologic grade*				0.318
Well differentiated	3 (1.8%)	3 (3.1%)	0 (0.0%)	
Moderate differentiated	154 (94.5%)	91 (93.8%)	63 (95.5%)	
Poorly differentiated	5 (3.1%)	2 (2.1%)	3 (4.5%)	
Undifferentiated	1 (0.6%)	1 (1.0%)	0 (0.0%)	
PET/CT parameters				
Tumor SUVmax	15.3 ± 7.7	14.3 ± 7.6	16.7 ± 7.7	0.041
Tumor MTV	24.1 ± 22.6	19.0 ± 16.9	31.4 ± 27.5	0.001
Nodal FDG uptake finding				<0.001
Negative	127 (76.5%)	92 (93.9%)	35 (51.5%)	
Positive	39 (23.5%)	6 (6.1%)	33 (48.5%)	
Nodal SUVmax	1.1 ± 2.5	0.2 ± 0.6	2.4 ± 3.5	<0.001

### Uni- and Multivariate Analyses

**Figure 1:**
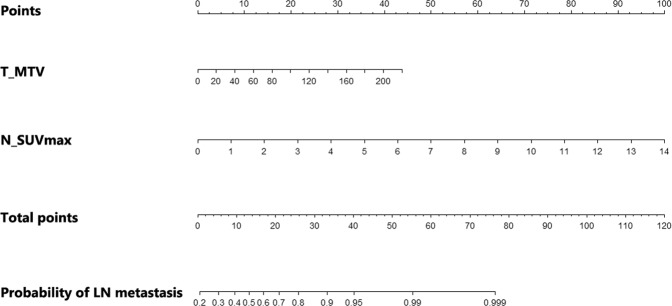
Nomogram for predicting the probability of regional LN metastasis using pretreatment [^18^F]FDG PET/CT parameters. First, the number of points for each parameter – T_MTV and N_SUVmax – should be determined by drawing a vertical line from the exact value of variables to the points row. Subsequently, total points can be obtained by sum of two variables. The individual predictive probability of regional LN metastasis can be calculated by drawing a vertical line from the total points row to the probability of regional LN metastasis. LN = lymph node; [^18^F]FDG = [^18^F]Fluorodeoxyglucose; PET/CT = Positron Emission Tomography/Computed Tomography; T_MTV = metabolic tumor volume of primary tumor; N_SUVmax = maximum standardized uptake value of regional LN.

Univariate logistic regression analysis revealed that T_SUVmax, T_MTV, and N_SUVmax were significantly associated with LN metastases (Table 2). In the multivariate analysis, both T_MTV (odds ratio [OR], 1.022; 95% confidence interval [CI], 1.001–1.043; *P* = 0.038) and N_SUVmax (OR, 2.181; 95% CI, 1.523–3.125; *P* < 0.001) were found to be significant predictive factors; otherwise, T_SUVmax was justifiably removed from the stepwise model. These two independent parameters were used to construct a nomogram for risk prediction of LN metastasis (Fig. 1). To use the nomogram, the points for each parameter should be determined by drawing a vertical line from the exact value of the variables to the points row. Then, total points are calculated by arithmetic sum. Finally, the individual predictive probability of LN metastasis can be obtained by drawing a vertical line from the total points row to the probability of LN metastasis row.

**Table 2:** Uni- and multivariate logistic regression analyses for regional lymph node metastases. OR = Odds Ratio; CI = Confidence Interval; CEA = Carcinoembryonic Antigen; PET/CT = Positron Emission Tomography/Computed Tomography; SUVmax = maximum standardized uptake value; MTV = Metabolic Tumor Volume.

Variables	Univariate Analysis		Multivariate Analysis	
	OR (95% CI)	P value	OR (95% CI)	P value
Age, years	0.991 (0.962–1.020)	0.538		
Sex				
Male				
Female	1.579 (0.845–2.951)	0.152		
Pre-operative CEA, ng/ml	0.998 (0.983–1.013)	0.785		
Pathologic tumor size, cm	1.024 (0.961–1.092)	0.463		
PET/CT parameters				
Tumor SUVmax	1.043 (1.001–1.088)	0.046		
Tumor MTV	1.033 (1.014–1.054)	<0.001	1.022 (1.001–1.043)	0.038
Nodal SUVmax	2.356 (1.637–3.392)	<0.001	2.181 (1.523–3.125)	<0.001

### Comparison of LN Metastasis Prediction Performance

**Figure 2:**
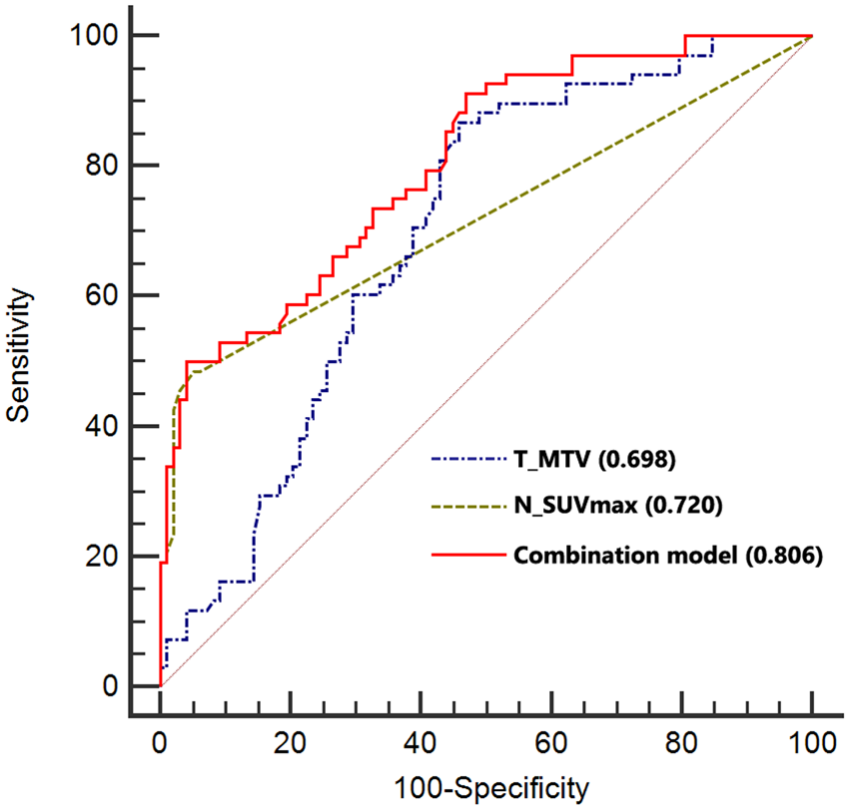
Graphs of ROC curve analysis show significant additional value of T_MTV for predicting regional LN metastasis in rectal cancer. Although comparison of AUC between N_SUVmax (0.720) and T_MTV (0.698) was not significant (*P* = 0.64), c-statistics when T_MTV was added to N_SUVmax (0.806) showed significant improvement in accuracy of risk prediction for LN metastasis (0.806 for c-statistics of combination model vs. 0.698 for T_MTV; *P* = 0.0002, 0.806 for c-statistics of combination model vs. 0.720 for N_SUVmax; *P* = 0.0008). ROC = receiver operating characteristic; T_MTV = metabolic tumor volume of primary tumor; LN = lymph node; AUC = area under the curve; N_SUVmax = maximum standardized uptake value of regional LN.

When LN metastases prediction performance was analyzed using receiver operating characteristic (ROC) curve analysis, the area under the curve (AUC) was 0.698 for T_MTV (Standard Error [SE], 0.040; 95% CI, 0.622–0.767) and 0.720 for N_SUVmax (SE, 0.033; 95% CI, 0.646–0.787). We were able to build an improved prediction model that combined T_MTV and N_SUVmax. The c-statistics of combined model was 0.806 (SE, 0.034; 95% CI, 0.737–0.863), and it showed significant improvement in accuracy of LN metastases prediction compared to T_MTV (*P* = 0.0002) or N_SUVmax (*P* = 0.0008) alone (Fig. 2). However, no significant difference was found between T_MTV and N_SUVmax (*P* = 0.64).

## Discussion

**Figure 3:**
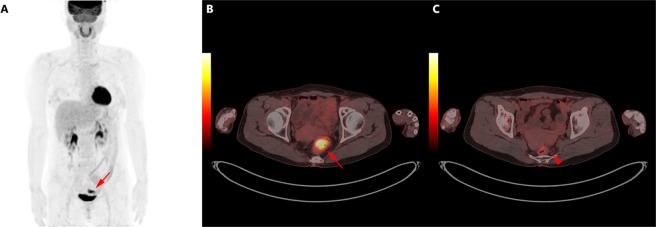
(**A**) Sixty-two-year-old female patient diagnosed with rectal cancer. (**B**) [^18^F]FDG PET/CT showed that intense [^18^F]FDG uptake in the rectum (T_SUVmax; 10.7, T_MTV; 13.7 ml). (**C**) Small LNs without significant [^18^F]FDG uptake were observed in the pericolic space, however, 3 of 24 resected LNs were histologically confirmed LN metastases. [^18^F]FDG = [^18^F]Fluorodeoxyglucose; PET/CT = Positron Emission Tomography/Computed Tomography; T_SUVmax = maximum standardized uptake value of primary tumor; T_MTV = metabolic tumor volume of primary tumor; LN = lymph node.

In the current study, we assessed the diagnostic value of metabolic parameters measured by [^18^F]FDG PET/CT for the prediction of LN metastases in patients with rectal cancer. Our results demonstrated that nodal [^18^F]FDG uptake findings were highly specific for LN metastases status, but it had a limitation due to its relatively low sensitivity. To overcome this limitation, we used metabolic parameters such as T_MTV and N_SUVmax for precise diagnosis of LN metastases. T_MTV and N_SUVmax are independent predictive factors for LN metastases in patients with rectal cancer. Moreover, the combination of both parameters significantly improved LN metastases prediction beyond each independent parameter alone (Fig. 3).

The sensitivity of [^18^F]FDG PET/CT was found to be relatively low (48.5%), although the specificity was high (93.9%). This finding is similar to that in previous studies, which showed poor sensitivity for detecting LN metastases^12,19^. In this study, we excluded the patients who had received neoadjuvant treatment, since any treatment before surgical resection could affect the histopathologic results, including the initial LN status. If these advanced rectal cancer patients who underwent neoadjuvant chemotherapy were included in the present study, the LN detectability of [^18^F]FDG PET/CT may be improved because the majority of these patients have shown high nodal [^18^F]FDG uptake.

Jo *et al*. have shown that MTV and total lesion glycolysis of the primary tumor, which are volumetric parameters of [^18^F]FDG PET/CT, were useful predictive factors for LN metastasis in patients with rectal cancer^16^. However, they only focused on metabolic activity of the primary tumor and did not use visual or semiquantitative information from [^18^F]FDG-avid LNs as a possible predictive factor for LN metastasis. In contrast, we evaluated not only metabolic information from the primary tumor, but also the LN’s own metabolic activity, for LN metastases prediction. For semiquantitative analysis of [^18^F]FDG-avid LNs, we adopted N_SUVmax which is the most widely accepted parameter of [^18^F]FDG PET/CT. Because SUVmax is a simple measurable metabolic parameter, and the application of metabolic volume parameter has a limitation in small volumes^20^. Tsunoda *et al*. demonstrated that N_SUVmax could improve the accuracy of preoperative LN metastases detection when compared to nodal diameter^12^. Concordantly, N_SUVmax was an independent prognostic factor for LN metastases in this study. We, therefore, have incorporated N_SUVmax as well as T_MTV for LN metastases prediction. Additionally, the combination of T_MTV and N_SUVmax could improve LN metastases prediction in patients with rectal cancer.

The present study also suggested that T_MTV could be a useful complementary predictive factor itself for LN metastases. Recently, several studies have shown that volumetric parameters measured by [^18^F]FDG PET/CT are useful for the evaluation of therapeutic response or prognostication in a variety of malignancies^21–25^; T_MTV can be used as a predictive factor for LN metastases in lung, endometrial, and uterine cervical cancers^18,26,27^. Our result is consistent with previous studies in that T_MTV obtained by [^18^F]FDG PET/CT could be an effective marker of total tumor burden and may reflect the aggressiveness of cancer associated with LN metastases. A previous meta-analysis identified numerous histopathological factors that may be correlated with LN metastases in primary CRC^28^. However, no single histopathological feature reliably predicted LN metastases, and these factors can be evaluated only after surgery. Considering the feasibility of [^18^F]FDG PET/CT in the pre-operative setting of rectal cancer, [^18^F]FDG PET/CT could be used as a non-invasive and pre-operative diagnostic tool for assessment of LN status in patients with rectal cancer.

The status of LN metastases is the most important prognostic factor in patients with rectal cancer. The five-year survival rate for patients with node-negative rectal cancer is 70–80%, whereas it is only 30–60% in node-positive rectal cancer. Accordingly, LN status assessment remains one of main factors used in determining neoadjuvant treatment in patients with rectal cancer^29^. Although [^18^F]FDG PET/CT can affect staging and treatment strategies, the current evidence is not considered strong enough to recommend the routine use of [^18^F]FDG PET/CT for initial staging^30,31^. The main reason for this is the lack of additional diagnostic value of [^18^F]FDG PET/CT. In that regard, the improved diagnostic performance of the combination model for predicting LN metastases maybe helpful in selecting patients who should receive neoadjuvant treatment. Additionally, [^18^F]FDG PET/CT might be helpful in determining risk-adapted treatment and long-term patient outcome by providing more accurate information regarding LN status. A multi-institutional, large prospective study is necessary for the combination model of T_MTV and N_SUVmax on pretreatment [^18^F]FDG PET/CT to be accepted as a significant prognostic factor in patients with rectal cancer.

This study had a few limitations. First, the single-center retrospective design of this study might be subject to selection bias. Further studies are needed to validate the results of the present study. Second, physiologic [^18^F]FDG uptake in the gastrointestinal tract may cause overestimation of T_MTV. Therefore, we adopted an absolute SUV threshold of 2.5 for MTV measurement, which was a widely used cutoff value in several previous studies and could reduce inter- and intra-observer variation in delineation of tumor volume using a software-assisted automatic method^21,32^. Lastly, we did not correct for a partial volume effect, which may have underestimated the value of SUVmax; this is because partial volume correction is generally too complex to use in daily clinical practice. New and feasible partial volume correction methods would be helpful in achieving precise quantification and clinical application^33,34^. Despite these limitations, we have shown that the combination of T_MTV and N_SUVmax could be an attractive strategy to further improve the diagnostic performance of [^18^F]FDG PET/CT for LN metastases in patients with rectal cancer.

In conclusion, T_MTV and N_SUVmax were independent prognostic factors for the prediction LN metastases in rectal cancer patients. Furthermore, our prediction model using T_MTV and N_SUVmax could provide a more precise prediction of LN metastases. The use of N_SUVmax in combination with T_MTV on preoperative [^18^F]FDG PET/CT could be a useful tool for initial staging and treatment planning in patients with rectal cancer.

## Methods

### Patients

Between January 2009 and August 2016, the medical records of 296 consecutive patients who underwent surgery for rectal cancer and had a preoperative [^18^F]FDG PET/CT were evaluated retrospectively. Of these, patients who received neoadjuvant chemoradiotherapy (n = 96), endoscopic tumor removal prior to surgery (n = 24), or long delayed interval over than 1 month between [^18^F]FDG PET/CT and surgery (n = 10) were excluded. A total of 166 patients were enrolled in this study. Surgery (160 patients with anterior resection, 4 with abdominoperineal resection, 1 with Hartmann’s operation, and 1 with total proctocolectomy) was performed by experienced colorectal surgeons, which included total mesorectal excision and at least 14 LNs were harvested. All of the patients were pathologically staged according to the 7th AJCC staging system^35^. The Institutional Review Board of Keimyung University Dongsan Medical Center approved this retrospective study and waived the requirement to obtain informed consent (2018-04-008).

### Positron Emission Tomography

[^18^F]FDG PET/CT scan was performed using an integrated PET/CT system (Discovery STe; GE Healthcare, Milwaukee, WI, USA or Biograph mCT; Siemens Medical Systems, Knoxville, TN, USA). All patients fasted for at least 6 hours before [^18^F]FDG injection, and the blood glucose levels were below 150 mg/dL. Patients were encouraged to rest during the [^18^F]FDG uptake period. Images were acquired 60 minutes after 5.5 MBq/kg (Discovery STe) or 4.0 MBq/kg (Biograph mCT) of FDG was administered intravenously. First, low-dose CT scan (Discovery STe; peak voltage of 120 kVp and slice thickness of 3.75 mm, Biograph mCT; peak voltage of 120 kVp and slice thickness of 3 mm) was acquired for attenuation correction. Immediately following the CT scan, PET scan was obtained with an acquisition time of 3 min per bed position for Discovery STe and 1.5 min per bed position for Biograph mCT in 3D mode. PET images were reconstructed using an ordered-subset expectation maximum iterative reconstruction algorithm.

### Image Analysis

[^18^F]FDG PET/CT images were retrospectively interpreted in consensus by two experienced nuclear medicine physicians. First, LN metastases status was visually assessed and categorized into one of two groups. The metastatic LNs were categorized as positive, which showed increased [^18^F]FDG uptake from the surrounding background activity on PET regardless of size on CT. Subsequently, the volume of interest (VOI) of the primary tumor and LNs were manually drawn, and T_SUVmax and N_SUVmax were measured only in patients with positive [^18^F]FDG uptake for semiquantitative analyses. We assigned the SUVmax as 0 to patients with negative [^18^F]FDG uptake of the primary tumor or LNs. The SUVmax was calculated using the following formula: SUVmax = maximum activity in the region of interest (MBq/g)/[injected dose (MBq)/body weight (g)]. T_MTV, obtained with an SUV threshold of 2.5, was used to define the VOI.

### Statistical Analysis

Numeric data are expressed as mean ± standard deviation. Univariate and multivariate logistic regression analyses were performed to identify significant variables associated with LN metastasis. The prediction model of LN metastases, with the combination of significant parameters, was developed by using multivariable logistic regression modeling. A nomogram was established to be a graphic representation of the LN metastases prediction model based on the result of multivariate logistic regression analysis. The additional value of risk prediction for LN metastases was evaluated using c-statistics, and DeLong method was used to compare the difference between the AUC^36^. Statistical analyses were performed using MedCalc for Windows, version 18.2.1 (MedCalc Software, Ostend, Belgium) and R version 3.4.3 software (http://www.r-project.org, R Foundation for Statistical Computing, Vienna, Austria). All *P* values < 0.05 were considered statistically significant.

## Data Availability

The datasets generated and/or analyzed during the current study are available from the corresponding author on reasonable request.

## References

[1] Siegel RL, Miller KD, Jemal A (2017). Cancer Statistics, 2017. CA Cancer J Clin.

[2] McDonald JR, Renehan AG, O’Dwyer ST, Haboubi NY (2012). Lymph node harvest in colon and rectal cancer: Current considerations. World J Gastrointest Surg.

[3] Glynne-Jones R (2017). Rectal cancer: ESMO Clinical Practice Guidelines for diagnosis, treatment and follow-up. Ann Oncol.

[4] Ganeshalingam S, Koh D-M (2009). Nodal staging. Cancer Imaging.

[5] Grubnic S, Vinnicombe SJ, Norman AR, Husband JE (2002). MR evaluation of normal retroperitoneal and pelvic lymph nodes. Clin Radiol.

[6] Cserni G (2003). Nodal staging of colorectal carcinomas and sentinel nodes. J Clin Pathol.

[7] Bipat S (2004). Rectal cancer: local staging and assessment of lymph node Involvement with endoluminal US, CT, and MR Imaging—a meta-analysis. Radiology.

[8] Brown G (2003). Morphologic predictors of lymph node status in rectal cancer with use of high-spatial-resolution MR imaging with histopathologic comparison. Radiology.

[9] Sanli Y (2012). The utility of FDG-PET/CT as an effective tool for detecting recurrent colorectal cancer regardless of serum CEA levels. Ann Nucl Med.

[10] Schneider DA (2016). Relative Value of Restaging MRI, CT, and FDG-PET Scan After Preoperative Chemoradiation for Rectal Cancer. Dis Colon Rectum.

[11] Heo SH, Kim JW, Shin SS, Jeong YY, Kang HK (2014). Multimodal imaging evaluation in staging of rectal cancer. World J Gastroenterol.

[12] Tsunoda Y, Ito M, Fujii H, Kuwano H, Saito N (2008). Preoperative Diagnosis of Lymph Node Metastases of Colorectal Cancer by FDG-PET/CT. Jpn J Clin Oncol.

[13] Tateishi U (2007). Non-enhanced CT versus contrast-enhanced CT in integrated PET/CT studies for nodal staging of rectal cancer. Eur J Nucl Med Mol Imaging.

[14] Marcus C (2016). JOURNAL CLUB: Value of Quantitative FDG PET/CT Volumetric Biomarkers in Recurrent Colorectal Cancer Patient Survival. AJR Am J Roentgenol.

[15] Kim SJ, Chang S (2015). Volumetric parameters changes of sequential 18F-FDG PET/CT for early prediction of recurrence and death in patients with locally advanced rectal cancer treated with preoperative chemoradiotherapy. Clin Nucl Med.

[16] Jo HJ, Kim SJ, Kim IJ, Kim S (2014). Predictive value of volumetric parameters measured by F-18 FDG PET/CT for lymph node status in patients with surgically resected rectal cancer. Ann Nucl Med.

[17] Kim BH (2015). High metabolic tumor volume and total lesion glycolysis are associated with lateral lymph node metastasis in patients with incidentally detected thyroid carcinoma. Ann Nucl Med.

[18] Husby JA (2015). Metabolic Tumor Volume on 18F-FDG PET/CT Improves Preoperative Identification of High-Risk Endometrial Carcinoma Patients. J Nucl Med.

[19] Lu Y-Y (2012). A systematic review and meta-analysis of pretherapeutic lymph node staging of colorectal cancer by 18F-FDG PET or PET/CT. Nucl Med Commun.

[20] Sprinz C (2018). Effects of blood glucose level on 18F fluorodeoxyglucose (18F-FDG) uptake for PET/CT in normal organs: an analysis on 5623 patients. Sci Rep.

[21] Lee JW (2014). Prognostic Value of Metabolic Tumor Volume and Total Lesion Glycolysis on Preoperative 18F-FDG PET/CT in Patients with Pancreatic Cancer. J Nucl Med.

[22] Liu J (2016). Prognostic Value of 18F-FDG PET/CT in Surgical Non-Small Cell Lung Cancer: A Meta-Analysis. Plos One.

[23] Ryu IS (2013). Prognostic Value of Preoperative Metabolic Tumor Volume and Total Lesion Glycolysis Measured by 18F-FDG PET/CT in Salivary Gland Carcinomas. J Nucl Med.

[24] Fendler WP (2013). Validation of Several SUV-Based Parameters Derived from 18F-FDG PET for Prediction of Survival After SIRT of Hepatic Metastases from Colorectal Cancer. J Nucl Med.

[25] Dos Anjos DA (2016). Semiquantitative Volumetry by Sequential PET/CT May Improve Prediction of Complete Response to Neoadjuvant Chemoradiation in Patients With Distal Rectal Cancer. Dis Colon Rectum.

[26] Crivellaro C (2012). 18F-FDG PET/CT can predict nodal metastases but not recurrence in early stage uterine cervical cancer. Gynecol Oncol.

[27] Kim D-H (2014). Metabolic parameters using 18F-FDG PET/CT correlate with occult lymph node metastasis in squamous cell lung carcinoma. Eur J Nucl Med Mol Imaging.

[28] Glasgow SC, Bleier JIS, Burgart LJ, Finne CO, Lowry AC (2012). Meta-analysis of Histopathological Features of Primary Colorectal Cancers that Predict Lymph Node Metastases. J Gastrointest Surg.

[29] Ong MLH, Schofield JB (2016). Assessment of lymph node involvement in colorectal cancer. World J Gastrointest Surg.

[30] Petersen RK, Hess S, Alavi A, Høilund-Carlsen PF (2014). Clinical impact of FDG-PET/CT on colorectal cancer staging and treatment strategy. Am J Nucl Med Mol Imaging.

[31] National Comprehensive Cancer Network. Rectal Cancer (Version I. 2018), https://www.nccn.org/professionals/physician_gls/pdf/rectal.pdf. Accessed April 16, 2018.

[32] Jo HJ, Kim SJ, Lee HY, Kim IJ (2014). Prediction of survival and cancer recurrence using metabolic volumetric parameters measured by 18F-FDG PET/CT in patients with surgically resected rectal cancer. Clin Nucl Med.

[33] Wallsten E, Axelsson J, Sundstrom T, Riklund K, Larsson A (2013). Subcentimeter Tumor Lesion Delineation for High-Resolution 18F-FDG PET Images: Optimizing Correction for Partial-Volume Effects. J Nucl Med Technol.

[34] Anouan KJ (2017). 18F-FDG-PET partial volume effect correction using a modified recovery coefficient approach based on functional volume and local contrast: physical validation and clinical feasibility in oncology. Q J Nucl Med Mol Imaging.

[35] Edge, S. *et al*. *AJCC cancer staging manual*. *7th ed*. (Springer, 2010).

[36] DeLong ER, DeLong DM, Clarke-Pearson DL (1988). Comparing the areas under two or more correlated receiver operating characteristic curves: a nonparametric approach. Biometrics.

